# Draft Genome Sequences of Two Cyanobacteria *Leptolyngbya* spp. Isolated from Microbial Mats in Miravalles Thermal Spring, Costa Rica

**DOI:** 10.1128/MRA.00553-21

**Published:** 2021-10-14

**Authors:** L. Brenes-Guillén, D. Vidaurre-Barahona, M. Mora-López, L. Uribe-Lorío

**Affiliations:** a Center for Research in Cellular and Molecular Biology, Universidad de Costa Rica, San José, Costa Rica; b School of Agronomy, Universidad de Costa Rica, San José, Costa Rica; University of Southern California

## Abstract

We report the draft genome sequences of *Leptolyngbya* sp. strain 7M and *Leptolyngbya* sp. strain 15MV, isolated from Miravalles Thermal Spring, Costa Rica. The thermophilic cyanobacteria exhibit unique diversity features that provide insight into the adaptation and evolution of phototrophic microorganisms in geothermal habitats.

## ANNOUNCEMENT

*Leptolyngbya* sp. is one of the most common filamentous cyanobacteria found in microbial mats in thermal springs (1, 2). Nevertheless, there are few genomes from tropical and thermal environments deposited in any databases. *Leptolyngbya* sp. strain 7M and *Leptolyngbya* sp. strain 15MV were isolated from a biofilm in Miravalles Thermal Spring, Costa Rica, in 2004 and 2012, respectively. They were cultivated in BG11 medium (3) and maintained at 25°C. The location, sampling methods, and isolation protocol details were the same as those provided in reference 4. Genomic DNA (gDNA) was isolated using the Plant II kit (Macherey-Nagel) and purified using the NucleoSpin gDNA cleanup kit (Macherey-Nagel), according to the manufacturer’s protocol. The total DNA was processed using a Nextera XT DNA library prep kit and MiSeq paired-end sequencing (MiSeq reagent kit v2; 2 × 250 bp; Illumina, Inc.) at the Center for Research in Cellular and Molecular Biology (CIBCM) of the University of Costa Rica. All the bioinformatics analyses were performed using the Kabré supercomputer, National High Technology Center (CeNAT), Costa Rica. Default parameters were used for all software unless otherwise specified. The sequence reads were filtered using Trimmomatic v0.36 (5) (SLIDINGWINDOW:4:20 and MINLEN of 100 bp), resulting in 753,167 and 544,121 high-quality reads. *De novo* genome assembly was performed using Unicycler v0.4.8 (6). The NCBI Prokaryotic Genome Annotation Pipeline (PGAP) was used to annotate the contigs for deposit at GenBank. The average nucleotide identity (ANI) was obtained using ChunLab’s online ANI calculator (7).

**FIG 1:**
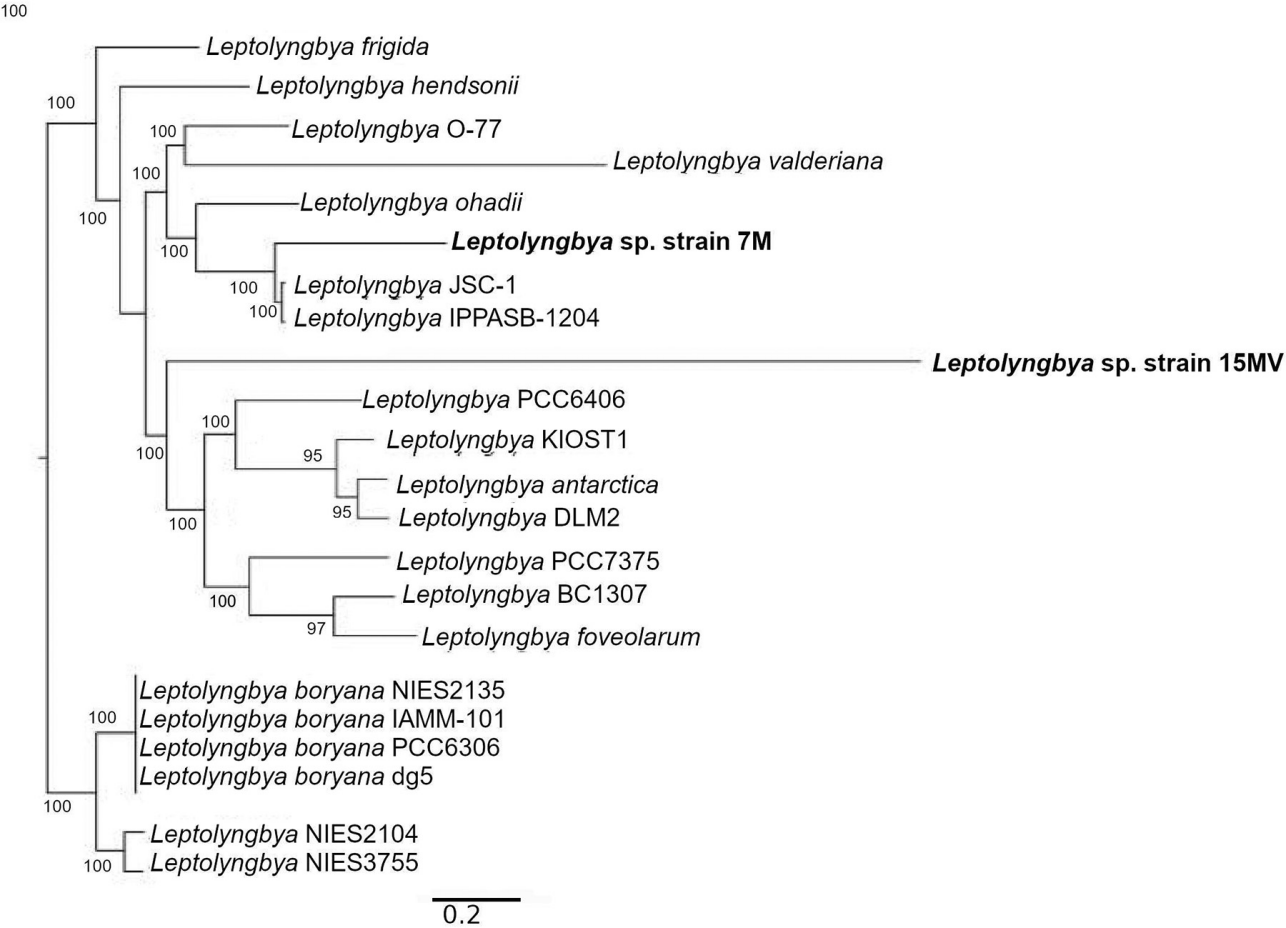
Phylogeny of 22 cyanobacteria based on a concatenated alignment of the protein sequences of 1,073 ortholog single-copy genes. Bootstrap values (%) are given at the nodes. The scale bar indicates the number of substitutions per site.

A core phylogeny was constructed using the protein sequences of the ortholog single-copy genes of *Leptolyngbya* sp. strains 7M and 15MV and published genome sequences derived from hot springs in Yellowstone National Park (8), Tolbo Lake, West Mongolia (9), a thermal spring in Aso-Kuju National Park, Japan (10), soil in Nitzana, Israel, mesophilic freshwater in California, and plankton in the Woods Hole region, MA, USA, using OrthoFinder v2.5.2 (11) (Fig. 1). The tree was inferred using Multiple Sequence Alignments (MSA) with the option “-M msa” (12). We used *Mu*ltiple *S*equence *C*omparison by *L*og-*E*xpectation (MUSCLE) to perform the multiple alignments of orthogroup sequences. FastTree was used for the maximum likelihood (ML) tree inference (13).

The draft genome sequence of *Leptolyngbya* sp. strain 7M was assembled into nonoverlapping scaffolds totaling 6,990,850 bp, with a G+C content of 49.92%, and it contains 7,020 coding sequences and 88 non-protein-coding genes; the final coverage was 10×. According to the 16S rRNA gene-based sequencing approach (4), isolate 7M was 98.59% identical to the siderophilic *Oscillatoriales* cyanobacterium JSC1. The draft genome sequence of *Leptolyngbya* sp. strain 15MV was assembled into nonoverlapping scaffolds totaling 5,089,511 bp, with a G+C content of 66.85%, and it contains 5,299 coding sequences and 86 non-protein-coding genes; the final coverage was 10×.

The average nucleotide identity (ANI)-based comparisons between the two strains was 96.80%; these genomes have low identity (<94% identity, query cover >95%) compared with genomes of *Leptolyngbya* spp. found in thermal springs and other environments. Based on the phylogeny, *Leptolyngbya* sp. strain 7M and *Leptolyngbya* sp. strain 15MV could be new species within the genera.

### Data availability.

The genome sequences of *Leptolyngbya* sp. strain 7M and *Leptolyngbya* sp. strain 15MV were deposited in DDBJ/ENA/GenBank under accession numbers CP070897.1 and CP071923.1, respectively, and BioProject accession number PRJNA702142; the raw data are publicly available under SRA accession numbers SRR14062498 and SRR14062499, respectively.
